# Challenges and opportunities in systems biology education

**DOI:** 10.1530/ERC-25-0024

**Published:** 2025-06-19

**Authors:** Jeff Didier, Sophia Croce, Salma Bayoumi, Elena Valceschini, Hugues Escoffier, Evelyn Gonzalez, Ali Kishk, Apurva Badkas, Sébastien De Landtsheer, Thomas Sauter

**Affiliations:** ^1^ Department of Life Sciences and Medicine, University of Luxembourg, Belvaux, Luxembourg; ^2^ Regional Student Group Luxembourg a.s.b.l., Student Council, International Society for Computational Biology, Esch-sur-Alzette, Luxembourg

**Keywords:** systems biology, higher education, master studies, alumni

## Abstract

Systems biology requires combining deep understanding in biology with technological methods and computational approaches to acquire new insights. Accordingly, students need to gain knowledge in very different disciplines and their integration to succeed in this truly interdisciplinary field. This review summarizes a variety of study lines at the master’s level and uses student and alumni feedback to highlight the main challenges and useful teaching approaches. Education in systems biology needs to be carefully designed to deliver deep knowledge in core aspects while still giving a broad overview of others. Teachers will need to find a good balance here. Integrated experimental and computational courses, as well as active learning approaches, can be key components of successful curricula. Training native systems biologists needs commitment by teachers and institutions and should start as early as possible.

## Introduction

Systems biology is a growing field of biological and biomedical research, driven by advancements in high-throughput sequencing technologies and the rapid rise of bioinformatics capabilities and machine learning. These advancements have enabled the collection and analysis of new types of biological data, allowing the completion of large-scale studies and, in turn, the formulation of new hypotheses. This development is reflected in a growing number of publications in the field, from 6,400 in 2003 to more than 35,000 in 2023 (scholar search, input ‘systems biology’). In recent years, the landscape of biomedical research was marked by many significant contributions such as the discovery of new RNA species functions (Memczak *et al.* 2013, Conn *et al.* 2015), the large-scale identification of protein structures (Jumper *et al.* 2021), and unprecedented insights into genomic and epigenetic regulation (Bujold *et al.* 2016).

This rapidly evolving landscape presents both opportunities and challenges for educational institutions. Beyond classical textbooks, traditional lab techniques, and standard analytical tools, both academic and industrial research increasingly demands not only the mastery of advanced biological concepts and computational skills, but also the capability to integrate these together in a ‘big picture’. Students and educators might be poorly prepared to materialize this type of systems thinking, given the qualitative nature of traditional biology courses and the scarcity of applied biology-related content in computer science curricula.

Addressing these needs, several scholars have proposed educational frameworks that integrate biology and advanced computational and modeling courses to adequately prepare students for the need of conceptualizing living systems in terms of their components, interactions, and emerging properties (Cvijovic *et al.* 2016, Anton Feenstra *et al.* 2018, Attwood *et al.* 2019, Jungck *et al.* 2020, Dale & Craig 2022, Momsen *et al.* 2022, Smith *et al.* 2022, Tamir *et al.* 2023). Some of them provide course structures or course design ideas, and most point to the fundamental difficulty of fostering mastery of complex biological phenomena, advanced computational techniques, and the ability to analyze systems as a whole beyond the sum of their components.

Several works have discussed the issues of systems biology education. For example, the Bulletin of Mathematical Biology discussed the challenges of cross-disciplinary educational requirements of math and biology students (Jungck *et al.* 2020). Feenstra *et al.* shared course structure and its evolution, learnings, and guidance for teachers based on the systems biology course conducted jointly by the Vrije Universiteit, Amsterdam, and the University of Amsterdam (Anton Feenstra *et al.* 2018). Momsen *et al.* discussed the systems framework that can translate classical biology instructions toward a systems biology approach, conceptualizing living systems in terms of their building blocks, underlying structure and function, along with their interactions with the environment, resulting in both outcomes and evolution of interacting entities (‘emergence’) (Momsen *et al.* 2022). In a preprint, Dale and Craig describe a successful combination of a study of a biochemical experiment (enzyme kinetics) with a coding exercise to demonstrate the applicability of modeling (Dale & Craig 2022).

More recent perspectives have broadened the vision of systems biology education, for example, advocating for expansion beyond biology into public health and computer science (Voit 2022), calling for more comprehensive computational goals and public outreach (Cvijovic *et al.* 2016, Voit *et al.* 2023), or summarizing developments in modeling approaches (Zupanic *et al.* 2020). While these works provide valuable high-level insight or conceptual guidance, they often focus on single institutional models, broad future agendas, or theoretical perspectives.

In this review, we aim to complement this literature by offering a grounded, comparative view of how systems biology education is currently implemented across a variety of institutions. We present a curated overview of program structures, highlight challenges based on direct input from students and alumni, and explore practical issues such as skill diversity, course prerequisites, interdisciplinary integration, AI tool adoption, and student well-being. Our goal is to bridge theory and practice, offering concrete recommendations that support a more adaptive and inclusive future for systems biology education.

## Examples of study lines

Most universities offer systems biology courses at the master’s level (Vilaprinyo *et al.* 2011), with a small but growing number of initiatives aimed at stimulating students’ interest in the discipline at the undergraduate level, especially during the COVID-19 period. Such initiatives include (hybrid) workshops, crash courses, or the introduction of Bachelor’s programs toward computational and systems biology offered by university departments, partnering societies, and graduate students (Mulder *et al.* 2018, LaTourrette *et al.* 2021). A variety of systems biology-oriented master’s programs are offered globally, either as specialization tracks or as fully entitled study lines. The main descriptive characteristics of these (offered for the academic year 2024/2025) have been sampled for multiple universities from European, North American, and Caribbean countries (Table 1). The term ‘systems biology’ is often hidden behind more complex program names, sometimes including the terms ‘bioinformatics’, ‘molecular’, ‘biochemistry’, or ‘computational biomedicine’. Other study lines directly refer to ‘systems biology’, either as a specialized track or as a fully titled program. The necessary credits and duration of the study lines are similar across most European countries with 120 ECTS over a 2-year period (corresponding to approximately 60 US semester credit hours or 240 CATS). There are some variations in countries such as France, England, the USA, and Cuba due to different educational systems, e.g., independent M1 and M2 levels in France over a 1-year period with 60 ECTS each, or 90 ECTS/180 CATS over the same period in England. The program structures typically focus on interdisciplinary approaches combining biology, bioinformatics, and computational methods. They focus on the inclusion of both theoretical and practical components while emphasizing individual research work and a thesis toward the end of the program duration. Nonetheless, the presented study lines show large variations in the way the programs are formatted. While some of them prefer problem- or project-based learning as a means of teaching, others offer a mix of lectures and seminars, including mandatory and elective units. As such, some programs can offer more flexibility in scheduling and thesis options, while others have a rather fixed curriculum and thesis track. The specialization tracks vary as well, with some study lines offering systems biology as a specialization, and others providing deeper specialization tracks such as cardiovascular, dynamic, neuro, or synthetic systems.

**Table 1:** Examples of systems biology master’s programs in European, North American, and Caribbean countries. The main characteristics of the different study lines leading to a Master’s degree in Systems Biology are displayed. MSc: Master of Science; ECTS: European Credit Transfer and Accumulation System; M2: second year in the French Master’s program system; and CATS: Credit Accumulation and Transfer Scheme.

Program name (degree)	Credits (duration)	Program structure	Specialization tracks
**University of Luxembourg (Esch-sur-Alzette, Luxembourg)**			
Molecular and Computational Biomedicine (MSc)	120 ECTS (2 years)	- Four semesters with around 15 weeks of full-time courses, plus additional time required for the exam preparation - The first semesters are course-based, mainly with block-courses of 2 weeks - The last 8 months individual research work is performed - The course content is around 1/3 lectures in biology and bio-medicine, 1/3 experimental and 1/3 computational practicals https://www.uni.lu/fstm-en/study-programs/master-in-molecular-and-computational-biomedicine	- Systems Biology - Biomedicine
**Maastricht University (Maastricht, Netherlands)**			
Systems Biology (MSc)	120 ECTS (2 years)	- Year 1, periods 1 and 2: problem-based learning followed by compulsory courses, depending on background: biology and physiology or mathematics of biological systems - Mandatory courses: systems biology, modeling biosystems, experimental design and data management - Periods 3 and 6: project I and project II - Periods 4 and 5: elective choices among omics, cardiovascular systems biology, or dynamical systems and non-linear dynamics, fundamental and systems neuroscience, modeling metabolism, or machine learning and multivariate statistics - Year 2, period 1: two elective courses from computational neuroscience, network biology, scientific programming, or commercialization and entrepreneurship - Periods 2–6: Master’s thesis https://curriculum.maastrichtuniversity.nl/education/master/systems-biology	- Omics - Cardiovascular Systems Biology - Dynamical Systems & Non-Linear Systems - Fundamental & Systems Neuroscience - Modeling Metabolism - Machine Learning & Multivariate Statistics
**Ghent University (Ghent, Belgium)**			
Bioinformatics (MSc)	120 ECTS (2 years)	- Common package (33 ECTS) of applied bioinformatics, including theoretical deepening and data analytical/problem-solving skills - Systems biology specialization module (28 ECTS) - Applied mathematics and informatics module (20 ECTS) - Optional courses (9 ECTS) https://studiekiezer.ugent.be/2023/master-of-science-in-bioinformatics-systems-biology-en	- Systems Biology - Bioscience Engineering - Engineering
**Université Paris-Saclay/Université d’Evry-Val-d’Essonne (Evry-Courcouronnes, France)**			
Systems and Synthetic Biology (M2 level) (MSc)	60 ECTS (1 year)	- Five core compulsory modules - Five selection modules (among 11) - Six-month research internship https://www.mssb.fr	- Systems Biology - Synthetic Biology
**Technical University of Denmark (Cophenhagen, Denmark)**			
Bioinformatics and Systems Biology (MSc)	120 ECTS (2 years)	- Polytechnical foundation courses (5 ECTS) - Program specific courses (55 ECTS, of which five in innovation courses, ten in mandatory courses, 40 in chosen program-specific courses) https://www.dtu.dk/english/education/graduate/msc-programmes/bioinformatics-and-systems-biology	- Biomedical Bioinformatics - Infectious Disease Health Informatics - Bioinformatic Methods in Life Sciences
**Imperial College London (South Kensington, UK)**			
Bioinformatics and Theoretical Systems Biology (MSc)	90 ECTS or 180 CATS (1 year)	- Composed of two core modules: bioinformatics and theoretical systems biology, and mathematics and computing - Computing project reinforcing programming skills through group project over 11 weeks - Bioinformatics and systems biology project applying course skills in research environment over 22 weeks - Lectures, computing labs, practical classes, presentations and seminars, group work - 30% projects; 70% examinations and coursework - Coursework, written exams, dissertation, computer and mathematics assignments - Individual research project, presentations, group report, and oral exam https://www.imperial.ac.uk/study/courses/postgraduate-taught/bioinformatics	No specialization tracks
**Karolinska Institute (Stockholm, Sweden)**			
Molecular Techniques in Life Science (MSc)	120 ECTS (2 years)	- First year advanced level courses in genetics and genomics, translational medicine, applied communication, and molecular life science methods, as well as the foundations of biostatistics, programming, bioinformatics, and comparative genomics - Second year mandatory courses in applied gene technology with bioinformatics analysis of large-scale data, and applied proteomics - The second half of the autumn semester offers three courses, of which the student should select two: systems biology, drug development, and a project course - During the spring semester, the individual degree project is performed https://education.ki.se/programme/5mt23-masters-programme-in-molecular-techniques-in-life-science	- Systems Biology - Drug Development
**University of Turku (Turku, Finland)**			
Molecular Systems Biology (MSc)	120 ECTS (2 years)	- Common courses - Track-specific major subject studies - Selectable studies - Master’s thesis https://www.utu.fi/en/study-at-utu/masters-degree-programme-in-biosciences-molecular-systems-biology	- Molecular Systems Biology - Evolutionary Biology
**University of Ottawa (Ottawa, Canada)**			
Biology (MSc)	60 ECTS (1 year)	- Multiple lecture and seminar available (63 in total) giving each 3 units/credits - No clear structure requirement given - Possible course components: lecture, theory and laboratory, magistral courses, and seminars https://catalogue.uottawa.ca/en/graduate/master-science-biology-specialization-bioinformatics	- Bioinformatics
**George Washington University School of Medicine and Health Sciences – Graduate School of Columbian College of Arts and Sciences (Washington, DC, USA)**			
Bioinformatics and Molecular Biochemistry (MSc)	30 credits (1–2 years)	- Thesis and non-thesis tracks - Eleven credits in required courses, 6 credits in required track, and 13 credits of electives (non-thesis option) or 6 credits in thesis and 7 credits of electives (thesis option) - Schedule flexibility, with 1-year or 2-year program option - Hands-on experience in a myriad of laboratories and research initiatives - Diverse elective course pool to choose from https://bulletin.gwu.edu/arts-sciences/biochemistry-molecular-medicine/ms-bioinformatics-molecular-biochemistry	- Cancer Biology - Inflammation - Pathobiology - Computational Genomics
**University of Havana (Havana, Cuba)**			
Biochemistry (MSc)	60 credits (3 years, hybrid)	- Basic mandatory courses: biomolecules, enzymology, immunology, biochemical method, metabolic biochemistry, research work - Research work evaluation: two research seminars in 4th and 5th semesters (8 credits each), pre-defense act before scientific council for thesis endorsement and writing (8 credits), remaining credits awarded upon thesis defense (8 credits) - Credit accumulation: minimum of 60 total credits required, maximum 24 credits per course (18 mandatory, 6 optional), 32 credits for research work, minimum 4 credits for non-lecture activities https://serviciosacademicos.fundacion.uh.cu/slides/maestria-en-bioquimica-91	- Biochemistry and Systems Biology - Immunology - Biotechnology - Molecular Biology

More detailed information on each study line, including the tuition fees, entry and language requirements, and application evaluations, can be reviewed in Supplementary Table S1 (see section on Supplementary materials given at the end of the article). Most strikingly among them, the total tuition fees accumulated over the study line period vary widely between national and international applicants, ranging from free in Sweden and Finland to around £15,000 GBP (€17,500 Euro) in England for national students, and from around €800 Euro in Luxembourg to nearly £37,000 GBP (€43,000 Euro) in England for international students. European universities typically have separate tuition fees for national and international students, while North American and Caribbean countries generally have higher tuition fees regardless of nationality. Entry requirements generally include a Bachelor’s degree from an accredited institution in life sciences or related fields, with a strong background in bioinformatics, for example, experience in coding, statistical analysis, work with large datasets, data visualization, or reproducibility practices. Some programs require a specific minimum number of credits in mathematics, molecular biology, and computer programming. Rarely, entry requirements may be linked to the student’s Bachelor’s passing grade (e.g., Ottawa, Canada, requiring at least 70% average score). Language requirements usually include a minimum CEFR English level of B1 to C1.

## How current students at University of Luxembourg see their studies

To also include student opinions in this review, we reached out to our students of the Master’s in Integrated Systems Biology (MISB) and of the International Master’s in Biomedicine (IMBM) at the University of Luxembourg. The MISB is currently renamed and slightly reoriented to the Master’s in Molecular and Computational Biomedicine (MMCB, Table 1). Both study lines – MISB/MMCB and IMBM – offer a 2-year full-time education consisting of 120 ECTS. All students spend the first semester together at the University of Luxembourg and receive experimental and computational training on genomics, transcriptomics, proteomics, and R programming, in addition to lectures on biology, biomedicine, and systems biology. We thereby follow a block-course structure of 2-week blocks per topic; see, for example, our project-based learning course on metabolic network modeling (Sauter *et al.* 2022). The use of 2-week thematic modules allows students to immerse themselves deeply in a focused topic, promoting both conceptual understanding and hands-on skill acquisition. One of the key motivations for this 2-week block-course structure is that it allows for regular laboratory practicals, which often require several uninterrupted hours and cannot be accommodated within traditional 90 min class slots. Moreover, it also facilitates close interaction with faculty and guest researchers and helps students see the integration of theory and practical application in a short, intensive timeframe. While the IMBM students then move to the partner universities in Strasbourg, France, and Mainz, Germany, for more training in biomedical and clinical research, the MISB/MMCB students stay in Luxembourg to deepen their knowledge in systems biology, other omics techniques, and molecular medicine. In addition, to specifically help biologists learn programming languages such as R, we use scaffolded exercises that start with biological questions and datasets familiar to them, progressively introducing statistical and scripting concepts. This is complemented by peer-assisted learning, interactive coding platforms, and workshop-style teaching where coding is integrated with data interpretation.

**Figure 1:**
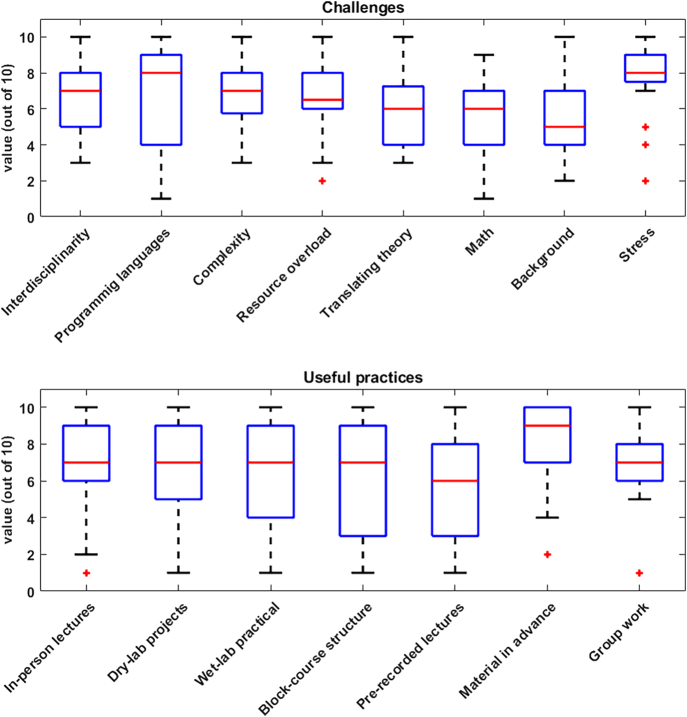
Feedback of current students of the Master’s in Integrated Systems Biology and the International Master’s in Biomedicine of the University of Luxembourg. Questionnaires were sent out to students in early 2024. The bar plots show the answers to the questions listed in Supplementary File S2.

In total, 21 students responded to the sent-out questionnaires (Fig. 1). When reporting about their main challenges, the feedback was quite heterogeneous, indicating more student-specific challenges rather than general issues. As most of our students are entering the master’s with training in biology, learning programming languages (starting with R) is challenging. In addition, the complexity of biological systems in general and the required interdisciplinarity and background, including in mathematics, were sometimes felt to be challenging. Together with an overload of available resources and topics to study, this often resulted in a very stressful start to the studies. This usually stabilizes when completing the coursework after the first year of the programs and when moving to the individual research-based second study year. But taking this feedback into account, the first semester is now simplified by making some courses optional and by adding two lecture-free weeks for digesting the studied material.

In addition, in terms of the useful practices, the student responses were heterogeneous (Fig. 1). Students appreciated most classical in-person lectures, computational dry-labs including project-based learning, and especially receiving the study material in advance, allowing them to prepare the courses in advance. Interestingly, pre-recorded lectures and group work in general were not always seen positively, the latter often due to very different backgrounds and learning paces of the group members. In addition, the block-course structure of our studies is sometimes seen critically as not allowing enough time to recap.

To furthermore focus on the challenges of students in bioinformatics when moving to research (e.g., master’s thesis), we organized within the ISCB-SC RSG Luxembourg association (International Society for Computational Biology Student Council’s Regional Student Group, see https://www.uni.lu/life-en/social-life/student-associations-clubs/rsg/) a dynamic graduate networking event titled ‘Struggles in Bioinformatics Education’ mostly with University of Luxembourg master’s and PhD students. As a result, the discussions highlighted a diverse landscape of challenges and opportunities for graduate students. These range from navigating the UNIX environment or a variety of programming languages, where Python emerges as a favored choice, to grappling with complex concepts such as Singularity containers or understanding the purpose and versatility of tools such as R Markdown. In terms of teaching methodologies, this extends to a clear preference for project-based learning, offering hands-on experience. However, students expressed concerns about the overwhelming workload, exacerbated by limited programming courses in Bachelor’s programs, which can make catching up difficult in master’s programs. In addition, questions arose about the availability of courses focusing on transferable skills, such as ‘good scientific practice’, ‘data visualization with python’, or ‘science communication’, that are only available to PhD students at the University of Luxembourg, highlighting a need for broader curriculum offerings.

## How alumni of University of Luxembourg assess their previous studies

**Figure 2:**
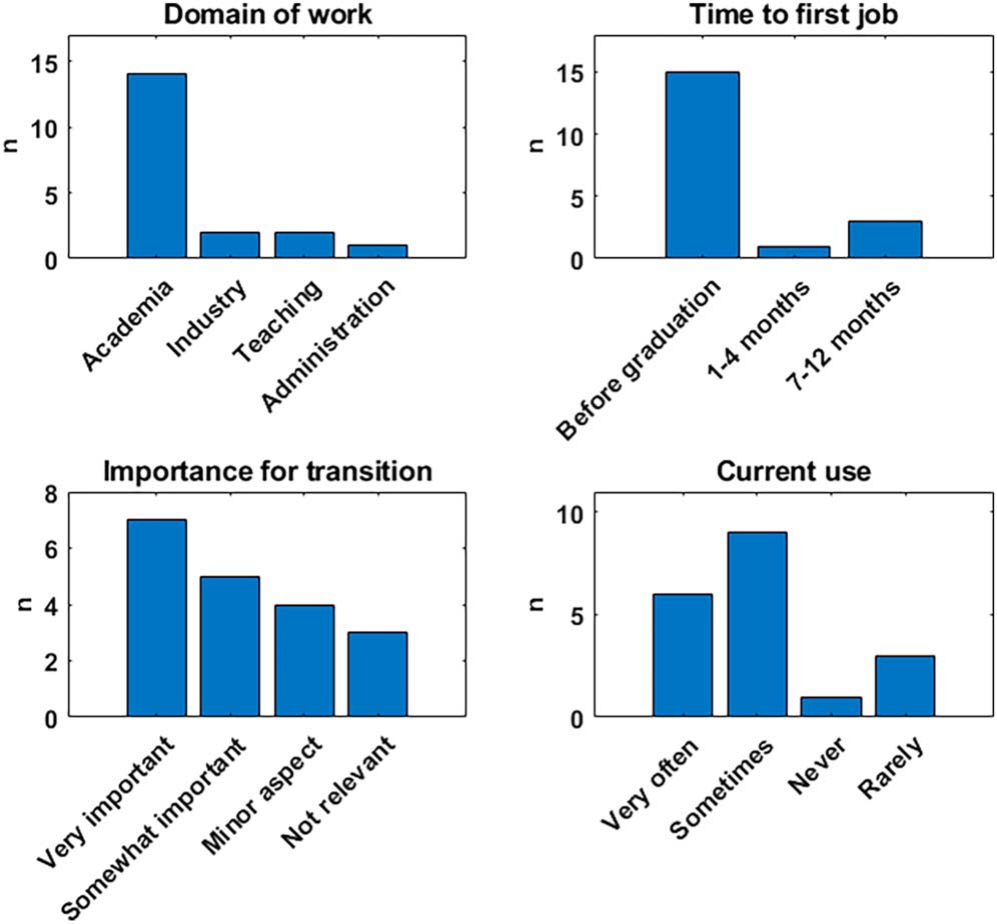
Feedback of alumni of the Master’s in Integrated Systems Biology and the International Master’s in Biomedicine of the University of Luxembourg. Questionnaires were sent out to alumni in 2024. The bar plots show the answers to the following questions (top left to bottom right): i) In which field are you working now? ii) How long did it take you to find your first employment after graduation? iii) How important were the systems biology/bioinformatics study courses for reaching your current job? iv) How often are you using systems biology/bioinformatics now?

To also get an overview of the long-term impact of the systems biology education, we reached out with a questionnaire to the alumni of the two master’s study lines at the University of Luxembourg connected to systems biology (Fig. 2). Most alumni who responded work in academia, with a few who moved to industry, teaching, or administration. Most found a job already before graduation and at the latest within 7–12 months after graduation. Most of the alumni considered the systems biology and bioinformatics aspects of their studies to be of high importance for their smooth transition to their current job. A vast majority of our alumni are currently using respective computational methods very often or at least sometimes, further underlying the high relevance of systems biology training. Interestingly, when assessing which methods are used by the alumni, a wide variety was indicated, ranging from analyzing different omics data types, images, and clinical data to metabolic and protein network modeling, dynamical pharmacokinetic modeling, and machine learning, as well as statistical approaches. In addition, programming in different languages such as R, Python, and MATLAB was mentioned. Programming in R is, for example, also used by alumni now teaching in schools and thus reaching pupils very early in their education, as well as other teachers. This overview illustrates the need for a broad introduction covering multiple subjects and methods in systems biology and bioinformatics. On the other hand, various topics were mentioned where deeper knowledge would be needed, ranging from programming to statistics, mathematics in general, and machine learning.

## Conclusions: challenges and opportunities

First, when reaching out to students via the course director or via student representatives, we have seen some differences in the feedback obtained. The emotionally more challenging points were mostly reported via the student representatives. As such, the student representatives form a vital link between students and institutions. They fulfill responsibilities such as relaying information, advocating for students, collecting feedback, and collaborating on solutions to enhance the overall student experience. Despite the importance of honest feedback, students may hesitate to provide it to their educators, opting instead to share more openly with their peers. This reluctance could stem from concerns about how expressing unfavorable feedback might impact their grades and assessments. In addition, the formal nature and inherent reverence in educator-student relationships may hinder students’ willingness to offer criticism. The possibility of providing feedback to peers in a less formal environment is very important.

From the feedback of students and alumni, as well as our own experience as teachers and study director, several key challenges in systems biology education can be identified and are summarized in the following sections.

## Interdisciplinarity

Systems biology at the master’s level attracts students of various disciplines – biology, biotechnology, pharmacy, as well as physics, engineering, and computer science, among other fields. While this is important to build up the needed interdisciplinarity, given such broad backgrounds, designing a curriculum and bringing everyone on a common ground is challenging. This has been recognized already in the literature on systems biology education, and several authors have provided guidelines and inputs for designing the courses. For example, Feenstra *et al.* have highlighted the need for depth and focus on core topics along with an overview of multiple related fields (Anton Feenstra *et al.* 2018). It is necessary to balance introducing core skills and competencies with fostering the ability to translate between different disciplines. Accordingly, it remains also an open question how and when the integration of students from different backgrounds is performed. It might be useful to have background-specific courses in the beginning, such as ‘math for biologists’ or ‘molecular biology for engineers’. Developing such foundational interdisciplinary courses can, however, be limited by faculty availability and expertise. Some institutions address this through co-teaching models, hiring interdisciplinary staff, or fostering collaborations between departments to pool expertise across domains. One approach adopted in other interdisciplinary programs to address varying student backgrounds is the use of pre-matriculation ‘boot camps’, typically held in the summer, which offer foundational training in key disciplines such as mathematics, biology, or computer science. These preparatory courses could be a valuable strategy for systems biology programs to help incoming students achieve baseline competency before formal coursework begins.

Establishing sustainable systems biology programs requires adequate infrastructure and teaching resources. While many computational tasks can be handled with standard laptops, more advanced analyses benefit from high-performance computing, reliable internet, and institutional IT support. Open-source software and cloud-based platforms can ease some of these challenges. Thus, some institutions do provide publicly available repositories with up-to-date teaching material and exercises. As an example, our systems biology curriculum includes a GitHub-based resource for the metabolic modeling course (https://github.com/sysbiolux/ISB705MetabolicNetworkModeling). Such resources not only reduce barriers to entry but also promote collaborative learning and curriculum development across institutions.

## Pre-requisites

On top of this, setting clear prerequisites for systems biology programs remains a challenge, particularly for students from non-quantitative backgrounds. At the University of Luxembourg, for example, while no strict coding threshold is enforced at admission, basic familiarity with a scripting language such as R or Python is highly recommended. To address varying entry levels, introductory programming sessions and self-paced resources are provided early in the curriculum, and some institutions have implemented preparatory boot camps to bridge foundational gaps.

During the above-mentioned RSG graduate networking event, participants also discussed potential solutions for some of these challenges. Pre-semester preparation, involving crash courses using Jupyter Notebooks, was highlighted to familiarize students with key concepts before the first semester. To promote deeper engagement, methods to encourage active learning were considered (Sauter *et al.* 2022, Sauter & Albrecht 2023) and avoiding superficial approaches, such as relying on prefilled scripts. In addition, there were proposals for workshops and training sessions aimed at providing foundational skills earlier in the academic journey, with interest in recording these sessions for accessibility. A yearly competition was also suggested to incentivize learning and collaboration through group projects, with potential prizes for participants. These proactive measures signify a commitment to enhance the learning experience and to prepare students for the rigors of bioinformatics.

## Systems medicine and translational research

A related question is how interdisciplinarity can be achieved. As Melissa Aikens stressed, ‘authentic problems are not simply putting a mathematics or physics problem in a biological context but rather applying principles from another discipline or bridging interdisciplinary concepts to solve questions that biologists answer’ (Jungck *et al.* 2020). This needs to start with the teachers, and even before, in the research environment of the institution. Compared to the early days of systems biology some 25 years ago, a lot has been achieved here already. Many research institutes and research projects are truly interdisciplinary and provide a great environment for the students to learn and become native systems biologists. Teaching also needs to become truly interdisciplinary. We need more integrated courses combining state-of-the-art knowledge with experimental and computational approaches. Students should seamlessly generate their own data, analyze these with computational approaches, and interpret the results in the context of the latest literature. This will not go without effort from the teacher’s side, but it is needed to foster interdisciplinarity. A notable development that reflects this interdisciplinary maturity is the emergence of systems medicine, where systems biology concepts are directly applied to clinical research and patient care. Programs such as those developed by the Institute for Systems Biology (https://isbscience.org/education-initiatives/systems-medicine-education/) and Georgetown University (https://systemsmedicine.georgetown.edu/) exemplify this shift, offering graduate-level training at the interface of biology, computation, and medicine. As the healthcare field increasingly incorporates systems-level approaches, it is likely that medical professionals will also require foundational knowledge in systems biology, presenting another dimension of opportunity – and responsibility – for systems biology education. In some European contexts, governmental support has been instrumental in developing and fostering such interdisciplinary programs, either through targeted funding initiatives, digitalization strategies, or infrastructure investment. For instance, national and EU-level grants have supported the creation of interdisciplinary graduate schools and infrastructure for data-intensive biology. However, the extent and type of support vary significantly across countries and institutions and are often influenced by national political priorities, such as investment in innovation, STEM education, or healthcare modernization. As such, the development and recruitment success of systems biology programs can be shaped by broader political agendas.

## Assessment challenges

Another challenge related to interdisciplinary learning is the difficulty of its assessment and the evaluation of systems thinking in students. As courses are typically graded by one final note, it is important to consider multiple techniques to ensure constructive evaluation across the disciplines involved. This can include rubrics for systems thinking by breaking down the exercises or projects into the specific disciplines and individually evaluating them (e.g., mathematics, coding, biological interpretation, critical thinking, and presentation skills). Another method would be peer evaluation and portfolio assessments of the students, where project progress is being documented and discussed. As an example, in our study line, students will be engaged in peer-evaluated project pitches and result presentations, but also classical exams, quizzes, and final assignments. Besides these methodologies of evaluating interdisciplinary learning, it remains important as a teacher to offer actionable feedback to the students and to make them aware of these opportunities.

## Standardization

It is also important to acknowledge that educational systems can vastly differ across countries, particularly when comparing the EU and the US. Undergraduate education in the US, for example, tends to be broader and more flexible, while European systems are often more specialized from early on. These differences can influence how systems biology programs are structured and what kind of preparation students bring with them. While most of the programs we listed are based in Europe, we also included examples from the US and tried to reflect these broader trends. Despite structural differences, a shared curriculum is still possible if it focuses on key competencies and learning outcomes, rather than enforcing a one-size-fits-all model. This would allow flexibility for institutions to adapt to the content while ensuring that core systems biology skills are covered.

## AI in systems biology education

Systems biology is a rapidly evolving field, and as our alumni mentioned, it is attractive and diverse in its applications (Fig. 2). This brings up the challenge of how to avoid being overwhelmed by the diversity and complexity of data and methods. Which resources to choose for learning, and how to condense and focus teaching? More specifically, it has often been shown to be challenging for students to learn new content within a course while at the same time learning how to write the necessary code. There must be enough time to deepen the knowledge. For this matter, the emergence of AI tools such as GitHub Copilot, ChatGPT, or other generative models has begun to influence coding education, including within systems biology. While we have not yet formally integrated these tools into the curriculum, students are increasingly using them informally to support coding tasks. This raises both opportunities – for personalized learning and error correction – and challenges, including overreliance or misuse. Further study is needed to define the best practices for integrating AI responsibly into scientific training, especially in systems biology education. On the other hand, for teachers, it might be challenging to include the fast evolution and development of new techniques. How to deal with this always-moving finishing line? One of the aspects here will be to start systems biology training during the Bachelor’s study or even earlier. As mentioned, some of our alumni are now teaching in school and thereby using R coding within their biology classes at the junior high-school level. This inspiring feedback points in the right direction.

## Big picture learning

And similarly, but in a longer perspective, how to develop and transmit a mindset of life-long learning? And how to balance study efforts and well-being? For example, at the University of Luxembourg, there is a growing effort of providing useful information and offers to the students toward improving mental health and well-being, including great self-help material (https://www.uni.lu/life-en/mental-health-wellbeing/). While it is important to see this emerge, it will be even more important in the long run to have these integrated within the studies from the beginning, not only when problems arise. Teachers will once again have to adopt it themselves first to be able to transmit it to their students.

Finally, despite systems biology being well received in the transition to the job market (Fig. 2), the gap between academia and industry might also be an issue, since the private sector typically uses more proprietary, unpublished methods and practices. Academic projects are typically limited, and often students need to be trained in long-term thinking in terms of public deliverables, as well as corporate practices and economics. This gap can be reduced by active academia-industry collaborations, also in the context of teaching.

In the face of these multifaceted challenges, the future of systems biology education lies in turning fragmentation into integration across disciplines, methodologies, and sectors to build a truly translational and future-ready educational paradigm. Embracing these challenges as opportunities will not only strengthen systems biology education but also ensure its relevance and resilience in a rapidly evolving scientific and societal landscape.

## Supplementary materials





## Declaration of interest

The authors declare that there is no conflict of interest that could be perceived as prejudicing the impartiality of the work reported.

## Funding

This work was supported by the Luxembourg National Research Fund (FNR) under Grant Nos. PRIDE17/12252781/DRIVEN, PRIDE21/16763386/CANBI02, and PRIDE21/16749720/NEXTIMMUNE2.

## Author contribution statement

TS designed the review. JD, SDL, AB, and TS contributed to the writing of the manuscript. All authors were involved in the collection and assessment of the data and approved the final version of the manuscript.
