# Supplementary material for: Challenges and opportunities in systems biology education
Source: Endocr Relat Cancer. 2025 Jun 19;32(6):e250024. doi: 10.1530/ERC-25-0024 (PMC12186286; doi:10.1530/ERC-25-0024)
Supplement: Supplementary file 2 [file supplementary_materials.pdf]

**Supplementary File S2:**

**Student questionnaire:**

- What background (Bachelor) did you have?

  Biological
  Computational
  Others (which?)

- Which Master are/were you enrolled in?

  MISB / IMBM

- Please rate how did you see the following from 1 (not at all challenging) to 10 (really challenging) during your studying:

  - Interdisciplinarity of system biology
    (need to have a broad knowledge in different subjects)

| | | | | | | | | | |
|---|---|---|---|---|---|---|---|---|---|
| 1 | 2 | 3 | 4 | 5 | 6 | 7 | 8 | 9 | 10 |

  - Learning a new programming language

| | | | | | | | | | |
|---|---|---|---|---|---|---|---|---|---|
| 1 | 2 | 3 | 4 | 5 | 6 | 7 | 8 | 9 | 10 |

  - Study the complexity of biological systems

| | | | | | | | | | |
|---|---|---|---|---|---|---|---|---|---|
| 1 | 2 | 3 | 4 | 5 | 6 | 7 | 8 | 9 | 10 |

  - Resource overload (navigate through the amount of online available resources)

| | | | | | | | | | |
|---|---|---|---|---|---|---|---|---|---|
| 1 | 2 | 3 | 4 | 5 | 6 | 7 | 8 | 9 | 10 |

  - Translate theory into practical application

| | | | | | | | | | |
|---|---|---|---|---|---|---|---|---|---|
| 1 | 2 | 3 | 4 | 5 | 6 | 7 | 8 | 9 | 10 |

  - Getting enough math and informatics basic knowledge

| | | | | | | | | | |
|---|---|---|---|---|---|---|---|---|---|
| 1 | 2 | 3 | 4 | 5 | 6 | 7 | 8 | 9 | 10 |

- Integrate your previous background (bachelor) with the different course requirements

| | | | | | | | | | |
|---|---|---|---|---|---|---|---|---|---|
| 1 | 2 | 3 | 4 | 5 | 6 | 7 | 8 | 9 | 10 |

- How stressful was your master's program? How frequently do you experience stress?

| | | | | | | | | | |
|---|---|---|---|---|---|---|---|---|---|
| 1 | 2 | 3 | 4 | 5 | 6 | 7 | 8 | 9 | 10 |

- <u>Please rate how did you find the following from 1 (not at all useful) to 10 (really useful) during your studying:</u>

  - In person theory lectures

| | | | | | | | | | |
|---|---|---|---|---|---|---|---|---|---|
| 1 | 2 | 3 | 4 | 5 | 6 | 7 | 8 | 9 | 10 |

- Hands on system biology/bioinformatics projects (Dry-lab projects)

| | | | | | | | | | |
|---|---|---|---|---|---|---|---|---|---|
| 1 | 2 | 3 | 4 | 5 | 6 | 7 | 8 | 9 | 10 |

- Biology wet lab practicals

| | | | | | | | | | |
|---|---|---|---|---|---|---|---|---|---|
| 1 | 2 | 3 | 4 | 5 | 6 | 7 | 8 | 9 | 10 |

- 2-week course structure

| | | | | | | | | | |
|---|---|---|---|---|---|---|---|---|---|
| 1 | 2 | 3 | 4 | 5 | 6 | 7 | 8 | 9 | 10 |

- Pre-recorded lectures

| | | | | | | | | | |
|---|---|---|---|---|---|---|---|---|---|
| 1 | 2 | 3 | 4 | 5 | 6 | 7 | 8 | 9 | 10 |

- Having the material (slides, guidelines, deadlines) in advance

| | | | | | | | | | |
|---|---|---|---|---|---|---|---|---|---|
| 1 | 2 | 3 | 4 | 5 | 6 | 7 | 8 | 9 | 10 |

- Group works

| | | | | | | | | | |
|---|---|---|---|---|---|---|---|---|---|
| 1 | 2 | 3 | 4 | 5 | 6 | 7 | 8 | 9 | 10 |
